# Effect of coronavirus disease 2019 (COVID‐19) on maternal, perinatal and neonatal outcome: systematic review

**DOI:** 10.1002/uog.22088

**Published:** 2020-07-01

**Authors:** J. Juan, M. M. Gil, Z. Rong, Y. Zhang, H. Yang, L. C. Poon

**Affiliations:** ^1^ Department of Obstetrics and Gynaecology Peking University First Hospital Beijing China; ^2^ Obstetrics and Gynecology Department, Hospital Universitario de Torrejón, Torrejón de Ardoz Madrid Spain; ^3^ School of Medicine Universidad Francisco de Vitoria (UFV), Pozuelo de Alarcón Madrid Spain; ^4^ Department of Gynaecology and Obstetrics, Tongji Hospital, Tongji Medical College Huazhong University of Science & Technology Wuhan China; ^5^ Department of Gynaecology and Obstetrics Zhongnan Hospital of Wuhan University Wuhan China; ^6^ Clinical Medicine Research Center of Prenatal Diagnosis and Birth Health in Hubei Province Wuhan China; ^7^ Department of Obstetrics and Gynaecology, Prince of Wales Hospital The Chinese University of Hong Kong Hong Kong SAR; ^8^ Harris Birthright Centre Fetal Medicine Research Institute, King's College Hospital, King's College London London UK

**Keywords:** coronavirus disease 2019, COVID‐19, neonatal outcome, pregnancy outcome, SARS‐CoV‐2, vertical transmission

## Abstract

**Objective:**

To evaluate the effect of coronavirus disease 2019 (COVID‐19) on maternal, perinatal and neonatal outcome by performing a systematic review of available published literature on pregnancies affected by COVID‐19.

**Methods:**

We performed a systematic review to evaluate the effect of COVID‐19 on pregnancy, perinatal and neonatal outcome. We conducted a comprehensive literature search using PubMed, EMBASE, the Cochrane Library, China National Knowledge Infrastructure Database and Wan Fang Data up to and including 20 April 2020 (studies were identified through PubMed alert after that date). For the search strategy, combinations of the following keywords and medical subject heading (MeSH) terms were used: ‘SARS‐CoV‐2’, ‘COVID‐19’, ‘coronavirus disease 2019’, ‘pregnancy’, ‘gestation’, ‘maternal’, ‘mother’, ‘vertical transmission’, ‘maternal–fetal transmission’, ‘intrauterine transmission’, ‘neonate’, ‘infant’ and ‘delivery’. Eligibility criteria included laboratory‐confirmed and/or clinically diagnosed COVID‐19, patient being pregnant on admission and availability of clinical characteristics, including at least one maternal, perinatal or neonatal outcome. Exclusion criteria were non‐peer‐reviewed or unpublished reports, unspecified date and location of the study, suspicion of duplicate reporting and unreported maternal or perinatal outcomes. No language restrictions were applied.

**Results:**

We identified a high number of relevant case reports and case series, but only 24 studies, including a total of 324 pregnant women with COVID‐19, met the eligibility criteria and were included in the systematic review. These comprised nine case series (eight consecutive) and 15 case reports. A total of 20 pregnant patients with laboratory‐confirmed COVID‐19 were included in the case reports. In the combined data from the eight consecutive case series, including 211 (71.5%) cases of laboratory‐confirmed and 84 (28.5%) of clinically diagnosed COVID‐19, the maternal age ranged from 20 to 44 years and the gestational age on admission ranged from 5 to 41 weeks. The most common symptoms at presentation were fever, cough, dyspnea/shortness of breath, fatigue and myalgia. The rate of severe pneumonia reported amongst the case series ranged from 0% to 14%, with the majority of the cases requiring admission to the intensive care unit. Almost all cases from the case series had positive computed tomography chest findings. All six and 22 cases that had nucleic‐acid testing in vaginal mucus and breast milk samples, respectively, were negative for severe acute respiratory syndrome coronavirus 2 (SARS‐CoV‐2). Only four cases of spontaneous miscarriage or termination were reported. In the consecutive case series, 219/295 women had delivered at the time of reporting and 78% of them had Cesarean section. The gestational age at delivery ranged from 28 to 41 weeks. Apgar scores at both 1 and 5 min ranged from 7 to 10. Only eight neonates had birth weight < 2500 g and nearly one‐third of neonates were transferred to the neonatal intensive care unit. There was one case of neonatal asphyxia and death. In 155 neonates that had nucleic‐acid testing in throat swab, all, except three cases, were negative for SARS‐CoV‐2. There were no cases of maternal death in the eight consecutive case series. Seven maternal deaths, four intrauterine fetal deaths (one with twin pregnancy) and two neonatal deaths (twin pregnancy) were reported in a non‐consecutive case series of nine cases with severe COVID‐19. In the case reports, two maternal deaths, one neonatal death and two cases of neonatal SARS‐CoV‐2 infection were reported.

**Conclusions:**

Despite the increasing number of published studies on COVID‐19 in pregnancy, there are insufficient good‐quality data to draw unbiased conclusions with regard to the severity of the disease or specific complications of COVID‐19 in pregnant women, as well as vertical transmission, perinatal and neonatal complications. In order to answer specific questions in relation to the impact of COVID‐19 on pregnant women and their fetuses, through meaningful good‐quality research, we urge researchers and investigators to present complete outcome data and reference previously published cases in their publications, and to record such reporting when the data of a case are entered into one or several registries. © 2020 The Authors. *Ultrasound in Obstetrics* & Gynecology published by John Wiley & Sons Ltd on behalf of the International Society of Ultrasound in Obstetrics and Gynecology.

## Abstract 2

This article's abstract has been translated into Spanish and Chinese. Follow the links from the abstract to view the translations.

## RESUMEN

**Efecto de la enfermedad coronavirus 2019 (COVID‐19) en el resultado materno, perinatal y neonatal: revisión sistemática**

**Objetivo:**

Evaluar el efecto de la enfermedad coronavirus 2019 (COVID‐19) en el resultado materno, perinatal y neonatal por medio de una revisión sistemática de la literatura publicada disponible sobre los embarazos afectados por COVID‐19.

**Métodos:**

Se realizó una revisión sistemática para evaluar el efecto de COVID‐19 en el resultado del embarazo, perinatal y neonatal. Se realizó una búsqueda exhaustiva de literatura utilizando PubMed, EMBASE, la Biblioteca Cochrane, la Base de Datos de la Infraestructura Nacional de Conocimiento de China y Wan Fang Data hasta el 20 de abril de 2020 inclusive (los estudios se identificaron mediante el sistema de alertas de PubMed después de esa fecha). Para la estrategia de búsqueda, se utilizaron combinaciones de las siguientes palabras clave y términos (en inglés) de Medical Subject Headings (MeSH): ‘SARS‐CoV‐2’, ‘COVID‐19’, ‘enfermedad coronavirus 2019’, ‘embarazo’, ‘gestación’, ‘materno’, ‘madre’, ‘transmisión vertical’, ‘transmisión materno‐fetal’, ‘transmisión intrauterina’, ‘neonato’, ‘bebé’ y ‘parto’. Los criterios de elegibilidad fueron COVID‐19 confirmado por laboratorio y/o diagnosticado clínicamente, el hecho de que la paciente estuviera embarazada en el momento del ingreso y la disponibilidad de características clínicas, con al menos un resultado materno, perinatal o neonatal. Los criterios de exclusión fueron los informes no revisados por pares o no publicados, la fecha y el lugar del estudio sin especificar, la sospecha de informes duplicados y los resultados maternos o perinatales no reportados. No se aplicaron restricciones de idioma.

**Resultados:**

Se identificó un elevado número de informes de casos y series de casos pertinentes, pero sólo 24 estudios, que incluían un total de 324 mujeres embarazadas con COVID‐19, cumplieron los criterios de elegibilidad y se incluyeron en la revisión sistemática. Estos comprendían nueve series de casos (ocho consecutivas) y 15 informes de casos. Los informes de casos se referían a un total de 20 pacientes embarazadas con COVID‐19 confirmado por un laboratorio. En los datos combinados de las ocho series de casos consecutivas, que incluían 211 (71,5%) casos de COVID‐19 confirmados por laboratorio y 84 (28,5%) casos diagnosticados clínicamente, la edad materna varió entre 20 y 44 años y la edad gestacional en el momento del ingreso fue entre 5 y 41 semanas. Los síntomas más comunes del cuadro clínico inicial fueron fiebre, tos, disnea/dificultad para respirar, fatiga y mialgia. La tasa de neumonía grave reportada en las series de casos varió entre el 0% y el 14%, y la mayoría de los casos requirieron el ingreso en la unidad de cuidados intensivos. Casi todos los casos de la serie de casos tuvieron resultados positivos en la tomografía axial computerizada. Los 6 y 22 casos en que se realizaron pruebas de ácido nucleico en muestras de mucosidad vaginal y leche materna, respectivamente, dieron negativo para el síndrome respiratorio agudo severo coronavirus 2 (SARS‐CoV‐2). Sólo se notificaron cuatro casos de aborto o interrupción del embarazo. En la serie de casos consecutivos, 219 de 295 mujeres habían dado a luz en el momento de la presentación del informe y el 78% de ellas lo hicieron mediante cesárea. La edad gestacional en el momento del parto varió entre 28 y 41 semanas. Las puntuaciones de Apgar a 1 minuto y a 5 minutos variaron entre 7 y 10. Sólo ocho recién nacidos pesaron al nacer <2500 g y casi un tercio de los recién nacidos fueron transferidos a la unidad de cuidados intensivos para recién nacidos. Hubo un caso de asfixia y muerte neonatal. Excepto tres casos, el resto de los 155 neonatos a los que se les hicieron pruebas de ácido nucleico en un frotis faríngeo, dieron negativo para el SARS‐CoV‐2. No hubo casos de muerte materna en las ocho series consecutivas de casos. Se reportaron siete muertes maternas, cuatro muertes fetales intrauterinas (una con embarazo de gemelos) y dos muertes de recién nacidos (embarazo de gemelos) en una serie no consecutiva de nueve casos con COVID‐19 grave. En los informes de casos se comunicaron dos muertes maternas, una muerte de recién nacido y dos casos de infección de recién nacidos por SARS‐CoV‐2.

**Conclusiones:**

A pesar del creciente número de estudios publicados sobre COVID‐19 en embarazos, no hay suficientes datos de buena calidad para sacar conclusiones no sesgadas con respecto a la gravedad de la enfermedad o a las complicaciones específicas de COVID‐19 en las mujeres embarazadas, así como a la transmisión vertical y a las complicaciones perinatales y neonatales. A fin de responder a preguntas específicas en relación con los efectos de COVID‐19 en mujeres embarazadas y sus fetos, mediante una investigación significativa de buena calidad, se insta a los investigadores y a los encargados de investigaciones a que presenten en sus publicaciones datos completos sobre los resultados y casos de referencia publicados anteriormente, y a que registren esos informes cuando los datos de un caso se introduzcan en uno o varios registros. © 2020 Los autores. Ultrasonido en Obstetricia y Ginecología publicado por John Wiley & Sons Ltd. en nombre de la Sociedad Internacional de Ultrasonido en Obstetricia y Ginecología.

## 摘要

**新型冠状病毒肺炎(COVID‐19)对孕产妇、围产期和新生儿结局的影响:系统性评价**

**目的:**

通过对有关新型冠状病毒肺炎(COVID‐19)影响的妊娠的已发表文献进行系统性评价来评估 COVID‐19 对孕产妇、围产期和新生儿结局的影响。

**方法:**

我们进行了系统性评价来评估COVID‐19对妊娠、围产期和

新生儿结局的影响。截至 2020 年 4 月 20 日(含)，我们使用 PubMed、EMBASE、Cochrane 图书馆、中国国家知识基础设施数据库和万方数据库

进行了全面文献检索(在 2020 年 4 月 20 日之后通过 PubMed 警报对这些研

究进行了识别)。将以下关键字和医学主题词(MeSH)术语的组合用作搜索策略:“SARS‐CoV‐2”、“COVID‐19”、“新型冠状病毒肺炎”、“妊娠”、“怀孕”、“孕产妇”、“母亲”、“垂直传播”、“母婴传播”、“宫内传播”、“新生儿”、“婴儿”和“分娩”。资格标准包括实验室确认和/或临床诊断的 COVID‐19、患者入院时已怀孕以及临床特征的可用性(包括至少一项孕产妇、围产期和新生儿结局)。排除标准为未经同行评议或未发表的报告、未指定研究的日期和地点、怀疑重复的报告以及未报告的母亲或围产期结局。未采用语言限制。

**结果:**

我们确定了大量相关的病例报告和病例系列，但是只有 24 项研究(包括总共 324 名患有 COVID‐19 的孕妇)符合资格标准而被纳入系统性评价中。其中包括 9 个病例系列(8 个连续)和 15 个病例报告。病例报告中，共包括 20 例经实验室确诊患有 COVID‐19 的孕妇。在 8 个连续病例系列的合并数据中(包括 211 例(71.5%)实验室确诊病例和 84 例(28.5%)临床确诊的 COVID‐19 病例)，孕产妇年龄为 20 至 44 岁，入院时胎儿胎龄为 5 到 41 周。有临床表现时的最常见症状是发烧、咳嗽、呼吸困难/呼吸急促、疲劳和肌痛。在病例系列中，报告的重症肺炎发生率为 0% 到 14%，大多数病例需要

在重症监护室接受治疗。病例系列中的几乎所有病例的计算机断层扫描胸部检查结果均为阳性。6 例和 22 例病例的阴道粘液和母乳样本接受了核酸检测，这些样本的严重急性呼吸系统综合征冠状病毒 2(SARS‐CoV‐2)结果均为阴性。仅报告了四例自然流产或终止妊娠的病例。在报告时，连续病例系列中，有219/295名孕妇分娩，其中78%接受了剖腹产。分娩时的胎龄范围为 28 至 41 周。1 分钟和 5分钟的阿普加评分范围为 7 到 10。只有八个新生儿的出生体重 <2500 g，并且近三分之一的新生儿被转入新生儿重症监护室。发生一例新生儿窒息死亡事件。接受了咽拭子核酸检测的 155 名新生儿中，只有三例病例的SARS‐CoV‐2检测结果为阳性。8 个连续病例系列中，没有出现孕产妇死亡病例。在 9 例 COVID‐19 非连续重症病例系列中，报告了 7 例孕产妇死亡、4 例宫内胎儿死亡(1 例双胎妊娠)和 2 例新生儿死亡(双胎妊娠)。在病例报告中，报告了 2 例孕产妇死亡、1 例新生儿死亡和 2 例新生儿 SARS‐CoV‐2 感染。

**结论:**

尽管有关患有 COVID‐19 的孕妇的已发表研究数量不断增加，但是在孕妇中 COVID‐19 严重程度或特定并发症、垂直传播、围产期和新生儿并发症方面，尚无足够的高质量数据可用于得出公正的结论。为了通过有意义的高质量研究来回答有关 COVID‐19 对孕妇及其胎儿的影响的具体问题，我们敦促研究者提供完整的结局数据并在其发表的研究中引用既往发表的病例，并在一个病例的数据被输入到一个或多个登记表时记录此类报告。© 2020 作者。威利父子公司(John Wiley & Sons Ltd)代表国际妇产科超声学会(ISUOG)出版《国际妇产超声杂志》(Ultrasound in Obstetrics & Gynecology)。

**CONTRIBUTION**

*What are the novel findings of this work?*

Based on consecutive case series, the rate of severe pneumonia in pregnant women with coronavirus disease 2019 (COVID‐19) was 0–14%; the majority of pregnancies were delivered by Cesarean section, there was one case of neonatal asphyxia and death, 155 neonates had nucleic‐acid testing in throat swabs and all, except three cases, were negative for severe acute respiratory syndrome coronavirus 2 (SARS‐CoV‐2). There were seven maternal deaths, four intrauterine fetal deaths and two neonatal deaths reported in a non‐consecutive case series of nine cases with severe COVID‐19. Amongst the case reports, two maternal deaths, one neonatal death and two cases of neonatal SARS‐CoV‐2 infection were reported.

*What are the clinical implications of this work?*

Despite the increasing number of published studies on COVID‐19 in pregnancy, there are insufficient good‐quality data to draw unbiased conclusions with regard to complications of COVID‐19 in pregnant women, as well as vertical transmission and perinatal complications. There is a need for good‐quality research. We urge researchers and investigators to present complete outcome data and reference previously published cases in their publications, and to record such reporting when the data of a case are entered into one or several registries.

## INTRODUCTION

With over 3 million individuals infected worldwide as of 2 May 2020^1^, the coronavirus disease 2019 (COVID‐19), caused by the severe acute respiratory syndrome coronavirus 2 (SARS‐CoV‐2), is a global public health crisis^2^, ^3^. Most cohort studies have focused on evaluating the effects of COVID‐19 on the general population^2^, ^3^, ^4^ and there are insufficient data on its impact on vulnerable populations, such as pregnant women.

It is recognized that pregnant women are at an increased risk of acquiring viral respiratory infection and developing severe pneumonia, due to the physiologic changes in their immune and cardiopulmonary systems^5^, ^6^. Lessons learned from the two previous notable coronavirus outbreaks, the severe acute respiratory syndrome coronavirus (SARS‐CoV) and the Middle East respiratory syndrome coronavirus (MERS‐CoV), suggest that pregnant women are particularly susceptible to adverse outcome, including the need for endotracheal intubation, admission to an intensive care unit (ICU), renal failure and death^7^, ^8^, ^9^. The first study describing the clinical characteristics and investigating the possibility of vertical transmission of SARS‐CoV‐2 in nine pregnant women, with laboratory‐confirmed COVID‐19, demonstrated that the severity of COVID‐19 in pregnant women was similar to that in non‐pregnant adults; and that there was no evidence of vertical transmission, as SARS‐CoV‐2 was not detected in amniotic‐fluid, cord‐blood and neonatal throat‐swab samples in six cases^10^. To date, the largest series reporting on both pregnancy and neonatal outcomes, including a total of 99 SARS‐CoV‐2‐infected pregnant women, demonstrated that COVID‐19 during pregnancy was not associated with an increased risk of adverse outcomes, such as spontaneous preterm birth^11^. None of the 100 neonates born to these women was infected with SARS‐CoV‐2. Based on very scarce data to date, conflicting evidence from nucleic acid‐based testing and antibody testing, in neonates born to mothers with COVID‐19, has raised further controversy in relation to the risk of vertical transmission during pregnancy^12^, ^13^.

The objective of this study was to perform a systematic review of available published literature on pregnancy affected by COVID‐19, in order to evaluate the effect of COVID‐19 on maternal, perinatal and neonatal outcome.

## METHODS

### Search strategy

We conducted a comprehensive literature search using PubMed, EMBASE, the Cochrane Library, China National Knowledge Infrastructure Database and Wan Fang Data, up to and including 20 April 2020 (studies were identified through PubMed alert after 20 April 2020). For the search strategy, combinations of the following keywords and medical subject heading (MeSH) terms were used: ‘SARS‐CoV‐2’, ‘COVID‐19’, ‘coronavirus disease 2019’, ‘pregnancy’, ‘gestation’, ‘maternal’, ‘mother’, ‘vertical transmission’, ‘maternal–fetal transmission’, ‘intrauterine transmission’, ‘neonate’, ‘infant’ and ‘delivery’. The search strategy is provided in Appendix S1.

Of note, at the peak of the SARS‐CoV‐2 outbreak in the Hubei province, China, cases with relevant symptoms, significant epidemiological history and typical computed tomography (CT) chest finding were clinically diagnosed as COVID‐19 pneumonia, without the need for laboratory confirmation. Therefore, eligibility criteria included laboratory‐confirmed and/or clinically diagnosed COVID‐19, patient being pregnant on admission and availability of clinical characteristics, including at least one maternal, perinatal or neonatal outcome. Exclusion criteria were non‐peer‐reviewed or unpublished reports, unspecified date and location of the study, suspicion of duplicate reporting and unreported maternal or perinatal outcomes. No language restrictions were applied.

### Study selection

Relevant titles were selected from the first screening and abstracts of citations were reviewed independently by two reviewers (J.J. and M.M.G.) to identify all potentially relevant articles. We identified a high number of case reports and case series. After the first screening of titles and abstracts, we decided to repeat the screening procedure and exclude case reports from China or case series that included fewer than 10 cases from China, in order to avoid duplication of cases as there have since been several cohort series published. The potentially relevant articles were evaluated by the same reviewers. The reference lists of the relevant original and review articles were searched for additional reports. Full‐text articles were retrieved for further consideration for inclusion. Disagreements were resolved by a third author (L.C.P.), who also reviewed independently the final included articles to confirm they met the inclusion criteria. The review was registered in PROSPERO on 23 April 2020, prior to data extraction (registration number: CRD42020181557)^14^.

### Data extraction, quality assessment and outcome measures

Two authors (J.J. and M.M.G.) extracted the information (population, outcome, study design and results) from the selected studies. A modified version of the Cochrane public health group data extraction and assessment template^15^, which was previously piloted by the researchers, was used to tabulate the findings of the included articles. The methodological quality of the studies was assessed independently by the same two authors using the Joanna Briggs Institute (JBI) tool for case series and case reports^16^. Publication bias was considered high since all the included studies were either case series or case reports. The quality of this review was validated using the preferred reporting items for systematic reviews and meta‐analyses (PRISMA) tool^17^.

The following information was extracted from the included studies: author names; institution and country; study design; sample size; maternal age; gestational age at admission; symptoms at admission; pregnancy complications; gestational age at delivery; mode of delivery; disease severity; laboratory and radiological findings; maternal and neonatal outcomes; and sample collection (amniotic fluid, cord blood, placenta, maternal vaginal secretion, urine, feces and breast milk, neonatal pharyngeal swab, neonatal blood, neonatal urine, neonatal feces, neonatal gastric juice). Any evidence of maternal–fetal transmission of SARS‐CoV‐2 was also recorded. Not all studies reported on all assessed variables. The denominators reported in the results were generated from the papers that provided such data. Studies that did not report on a specific outcome were recorded as not reported. We contacted directly the corresponding authors of potentially eligible studies, when further clarification on their data was needed; such as overlapping cases in different studies published by the same group or to complete outcome data. Details of the requested data and responses of the authors, or lack of response, are provided in Table S1.

### Statistical analysis

Due to the lack of studies with a design that would allow us to perform a meta‐analysis, we opted to perform a narrative synthesis using the Synthesis Without Meta‐analysis (SWiM) reporting guideline (intended to complement the PRISMA guidelines in such cases)^18^. Summary statistics (*n* (range)) were calculated to show sample distribution, where appropriate including only studies in which a consecutive (all cases) cohort had been reported.

## RESULTS

**Figure 1:**
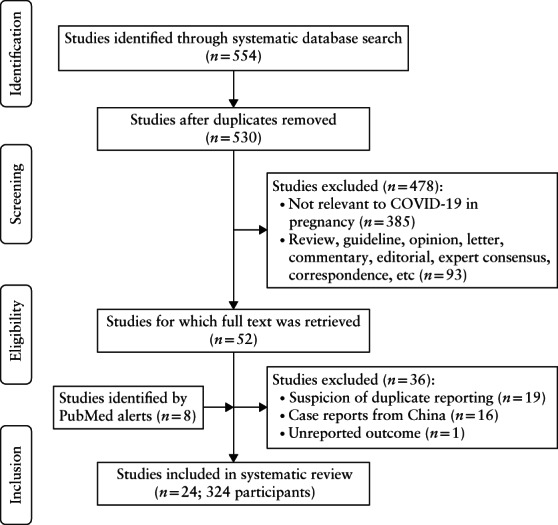
Flowchart showing inclusion in systematic review of studies reporting on pregnancies affected by COVID‐19.

Figure 1 summarizes the selection of articles for inclusion in the systematic review. The initial database search identified 554 records, which, after exclusion of duplicates, were screened for the eligibility criteria. Of 52 studies assessed in full text, 36 were excluded due to overlapping cases, being case reports from China or missing outcome data (Table S2). PubMed alerts were reviewed daily until submission and contributed eight additional studies. Two‐thirds of papers in the Chinese language were identified through both PubMed and Chinese databases. Finally, 24 studies, including a total of 324 pregnant women with COVID‐19, met the eligibility criteria and were included in the review. These comprised nine case series^11^, ^19^, ^20^, ^21^, ^22^, ^23^, ^24^, ^25^, ^26^, published between 4 March 2020 and 28 April 2020, and 15 case reports^27^, ^28^, ^29^, ^30^, ^31^, ^32^, ^33^, ^34^, ^35^, ^36^, ^37^, ^38^, ^39^, ^40^, ^41^, published between 25 March 2020 and 28 April 2020 (Table 1). Of the nine included case series, eight were consecutive and reported on a total of 295 pregnant patients^11^, ^19^, ^21^, ^22^, ^23^, ^24^, ^25^, ^26^. Of these, 211 (71.5%) cases had laboratory‐confirmed and 84 (28.5%) had clinically diagnosed COVID‐19. The 15 case reports^27^, ^28^, ^29^, ^30^, ^31^, ^32^, ^33^, ^34^, ^35^, ^36^, ^37^, ^38^, ^39^, ^40^, ^41^ included a total of 20 pregnant patients with laboratory‐confirmed COVID‐19.

**Table 1:** Study characteristics and maternal symptoms of COVID‐19 in case series and case reports included in systematic review

				Diagnosis			Symptom							
Study	*N*	Country	MA (years)	Laboratory	Clinical	GA on admission (weeks)	Fever	Cough	Fatigue	Dyspnea/shortness of breath	Sore throat	Myalgia	Malaise	Diarrhea/GI symptoms
*Case series*														
Breslin (2020)^19^	43	USA	20–39	43	0	NR	14	19	0	7	0	11	0	5
Ferrazzi (2020)^26^	42	Italy	21–44	42	0	NR	20	18	0	8	0	7	0	2
Hantoushzadeh (2020)^20^	9	Iran	25–49‡	9	0	24–36	9	9	0	6	1	0	0	0
Liu (2020)^21^	41	China	22–42	16	25	22–40	16	15	5	5	0	0	0	0
Liu (2020)^22^	13	China	22–36	13	0	NR	10	2	4	3	1	0	0	0
Liu (2020)^23^	15	China	23–40	15	0	12–38	13	9	4	1	1	3	0	1
Liu (2020)^24^	19	China	26–38	10	9	NR	11	5	0	5	0	0	0	2
Wu (2020)^25^	23	China	21–37	19	4	6–40	4	6	0	0	0	0	0	0
Yan (2020)^11^	99	China	24–41	53	46	5 to 41 + 2	50	27	15	10	8	6	0	1
Total*	295	—	20–44	211/295 (71.5)	84/295 (28.5)	5–41	138/295 (46.8)	101/295 (34.2)	28/295 (9.5)	39/295 (13.2)	10/295 (3.4)	27/295 (9.2)	0	11/295 (3.7)
*Case reports*														
Alonso Díaz (2020)^27^	1	Spain	41	1	0	38 + 4	0	1	0	1	0	0	0	0
Alzamora (2020)^28^	1	Peru	41	1	0	33	1	0	1	1	0	0	1	0
Buonsenso (2020)^29^, †	4	Italy	31–34	4	0	17, 24, 35, 38	3	4	0	1	0	0	0	0
Gidlöf (2020)^30^	1	Sweden	34	1	0	36 + 2	1	0	0	0	0	0	1	0
González Romero (2020)^31^	1	Spain	44	1	0	29 + 2	1	1	0	0	1	0	0	0
Iqbal (2020)^32^	1	USA	34	1	0	39	1	1	0	0	0	1	0	0
Juusela (2020)^33^	2	USA	26, 45	2	0	33 + 6, 39 + 2	0	0	0	1	0	0	0	1
Kalafat (2020)^34^	1	Turkey	32	1	0	35 + 3	1	1	0	1	0	0	0	0
Karami (2020)^35^	1	Iran	27	1	0	30 + 3	1	1	0	0	0	1	0	0
Kelly (2020)^36^	1	USA	NR	1	0	33	1	1	0	0	0	0	0	1
Lee (2020)^37^	1	Korea	28	1	0	37 + 6	1	1	0	0	1	0	0	0
Lowe (2020)^38^	1	Australia	31	1	0	40 + 2	1	1	0	0	0	0	0	0
Schnettler (2020)^39^	1	USA	39	1	0	31 + 0	1	1	0	1	0	0	1	0
Vlachodimitropoulou Koumoutsea (2020)^40^	2	Canada, France	23, 40	2	0	35 + 5, 35 + 2	2	2	0	0	0	0	0	0
Zamaniyan (2020)^41^	1	Iran	22	1	0	32	1	1	0	1	0	1	0	1

Only first author is given for each study.

Data are presented as *n*, range or *n*/*N* (%) unless indicated otherwise.

* Hantoushzadeh (2020)^20^ excluded from total calculations because not consecutive case series.

† Additional information for this study retrieved from Buonsenso (2020)^53^.

‡ ± 5 years for reasons of confidentiality.

GA, gestational age; GI, gastrointestinal; MA, maternal age; NR, not reported.

We recorded data related to maternal and perinatal characteristics, and the clinical manifestations of COVID‐19 at admission, including laboratory testing, treatment received and maternal, perinatal and neonatal outcome. The majority of the cases originated from China^11^, ^21^, ^22^, ^23^, ^24^, ^25^, but cases from Australia (one case report)^38^, Canada and France (one case report)^40^, Korea (one case report)^37^, Iran (two case reports and one case series)^20^, ^35^, ^41^, Italy (one case series and one case report)^26^, ^29^, Peru (one case report)^28^, Spain (two case reports)^27^, ^31^, Sweden (one case report)^30^, Turkey (one case report)^34^ and USA (one case series and four case reports)^19^, ^32^, ^33^, ^36^, ^39^ were also included. We differentiated cases that were laboratory‐confirmed from those that were diagnosed clinically, but data from the two types of diagnosis were combined for presentation. A laboratory‐confirmed case of COVID‐19 was defined as a positive result on quantitative reverse transcriptase polymerase chain reaction (qRT‐PCR) assay of maternal pharyngeal swab specimens. As mentioned, at the peak of the COVID‐19 outbreak in Hubei province, China, cases with relevant symptoms, significant epidemiological history and typical chest CT finding were diagnosed clinically as COVID‐19, because the viral nucleic acid test was reported^42^ to have a false‐negative rate of 30%.

Quality assessment of the included studies is reported in Tables S3 and S4. The inclusion of eight case series with consecutive cohorts partly mitigated the high publication bias expected from case reports.

### Clinical characteristics

In the combined data from the eight consecutive case series^11^, ^19^, ^21^, ^22^, ^23^, ^24^, ^25^, ^26^, the maternal age ranged from 20 to 44 years and the gestational age on admission ranged from 5 to 41 weeks (Table 1). The most common symptoms at presentation were fever, cough, dyspnea/shortness of breath, fatigue and myalgia. Based on the case series by Yan *et al*.^11^ and Breslin *et al*.^19^, up to one‐third of pregnant patients with COVID‐19 were asymptomatic on admission. The rate of severe pneumonia reported amongst the consecutive case series ranged from 0% to 14%, with the majority of the cases requiring ICU admission, of which only a few cases received invasive mechanical ventilation (Table 2). The majority of cases (70.7%) received antibiotic therapy, whilst only 82 of 217 (37.8%) and 31 of 176 (17.6%) cases received antiviral therapy and corticosteroids, respectively. Only two cases were reported to have received hydroxychloroquine. There were no cases of maternal death in the consecutive case series. Seven maternal deaths were reported in the Iranian case series^20^ of nine non‐consecutive cases with severe COVID‐19.

**Table 2:** Treatment and outcome of pregnant women with COVID‐19 reported in case series and case reports included in systematic review

			Treatment								
Study		*N*	Antiviral therapy	Antibiotic therapy	Corticosteroid	Hydroxychloroquine	Invasive mechanical ventilation	Non‐invasive ventilation	ICU admission	Severe pneumonia	Maternal mortality
*Case series*											
Breslin (2020)^19^		43	0	2	0	2	0	3	2	6	0
Ferrazzi (2020)^26^		42	NR	NR	NR	NR	7‡		4	NR	0
Hantoushzadeh (2020)^20^		9	9	9	0	6	9	0	9	9	7
Liu (2020)^21^		41	14	NR	NR	NR	NR	NR	0	0	0
Liu (2020)^22^		13	NR	NR	NR	NR	1	0	1	1	0
Liu (2020)^23^		15	11¶	15	0	0	0	14	0	0	0
Liu (2020)^24^		19	6	NR	0	NR	NR	NR	NR	NR	0
Wu (2020)^25^		23	NR	NR	NR	NR	NR	NR	NR	0	0
Yan (2020)^11^		99	51	94	31	NR	2	4	5	5	0
Total*		295	82/217 (37.8)	111/157 (70.7)	31/176 (17.6)	2/58 (3.4)	3/170§ (1.8)	21/170§ (12.4)	12/253 (4.7)	12/234 (5.1)	0
*Case reports*											
Alonso Díaz (2020)^27^		1	NR	NR	NR	NR	1	0	1	1	0
Alzamora (2020)^28^		1	1	1	1	1	1	0	1	1	0
Buonsenso (2020)^29^, †		4	4	0	0	4	0	1	1	1	0
Gidlöf (2020)^30^		1	0	1	0	0	0	1	0	0	0
González Romero (2020)^31^		1	1	1	1	1	1	0	1	1	0
Iqbal (2020)^32^		1	0	0	0	0	0	0	0	0	0
Juusela (2020)^33^		2	0	1	1	1	1	1	1	2	0
Kalafat (2020)^34^		1	1	1	1	1	1	0	1	1	0
Karami (2020)^35^		1	1	1	1	1	1	0	1	1	1
Kelly (2020)^36^		1	NR	NR	NR	NR	1	0	1	1	0
Lee (2020)^37^		1	0	0	0	0	0	0	0	0	0
Lowe (2020)^38^		1	0	1	0	0	0	0	0	0	0
Schnettler (2020)^39^		1	1	1	1	1	1	0	1	1	0
Vlachodimitropoulou Koumoutsea (2020)^40^		2	0	1	0	0	NR	NR	0	0	0
Zamaniyan (2020)^41^		1	1	1	1	1	1	0	1	1	1

Only first author is given for each study.

Data are presented as *n* or *n*/*N* (%).

* Hantoushzadeh (2020)^20^ excluded from calculation because not consecutive case series.

† Additional information for this study retrieved from Buonsenso (2020)^53^.

‡ Seven cases received oxygen support (nasal cannula, continuous positive airway pressure); authors did not specify invasive or non‐invasive ventilation.

§ Cases from Ferrazzi (2020)^26^ not included.

ICU, intensive care unit; NR, not reported.

Almost all women in the case series had positive CT chest findings, including patchy shadowing or ground‐glass opacity. On admission, the majority of the cases with laboratory test results had normal or low leukocyte count (146/182; 80.2%), while just under half had lymphocytopenia (85/197; 43.1%) and increased C‐reactive protein (CRP) (90/197; 45.7%) (Table 3). Of note, all six cases that had nucleic‐acid testing in vaginal mucus and all 22 cases that had nucleic‐acid testing in breast milk samples were negative for SARS‐CoV‐2.

**Table 3:** Radiological, laboratory and biological sample findings of pregnant women with COVID‐19 reported in case series and case reports included in systematic review

		Chest CT		Laboratory									Biological sample	
Study	*N*	Positive*	Negative	Leukocyte (normalor low)	Lymphocyte (low)	CRP (high)	AST/ALT (high)	Platelet (low)	Ferritin (high)	D‐dimer (high)	IgG (high)	IgM (high)	Vaginal mucus	Breast milk
*Case series*														
Breslin (2020)^19^	43	NR	NR	NR	NR	NR	NR	NR	NR	NR	NR	NR	NR	NR
Ferrazzi (2020)^26^	42	NR	NR	26	6	17	5	NR	NR	NR	NR	NR	NR	NR
Hantoushzadeh (2020)^20^	9	9	0	5	8	9	6	2	NR	NR	NR	NR	NR	NR
Liu (2020)^21^	41	38	3	24	25	27	NR	NR	NR	NR	NR	NR	NR	NR
Liu (2020)^22^	13	NR	NR	NR	NR	NR	NR	NR	NR	NR	NR	NR	NR	NR
Liu (2020)^23^	15	15	0	NR	12	10	NR	NR	NR	NR	NR	NR	NR	NR
Liu (2020)^24^	19	19	0	NR	NR	NR	NR	NR	NR	NR	NR	NR	NR	10; all negative
Wu (2020)^25^	23	23	0	NR	NR	NR	NR	NR	NR	NR	NR	NR	NR	NR
Yan (2020)^11^	99	88	4†	96	42	36	NR	NR	NR	NR	NR	NR	6; all negative	12; all negative
Total‡	295	183/190 (96.3)	7/190 (3.7)	146/182 (80.2)	85/197 (43.1)	90/197 (45.7)	5/42 (11.9)	NR	NR	NR	NR	NR	—	—
*Case reports*														
Alonso Díaz (2020)^27^	1	NR	NR	NR	NR	NR	NR	NR	NR	NR	NR	NR	NR	NR
Alzamora (2020)^28^	1	1	0	0	1	1	0	1	1	0	1	1	NR	NR
Buonsenso (2020)^29^, §	4	NP	—	4	4	4	NR	NR	NR	NR	NR	NR	NR	2; 1 negative, 1 positive
Gidlöf (2020)^30^	1	1	0	0	0	1	0	0	NR	NR	NR	NR	1; negative	1; negative
González Romero (2020)^31^	1	NR	NR	0	1	1	1	0	NR	1	NR	NR	NR	NR
Iqbal (2020)^32^	1	1	0	0	1	1	0	0	NR	1	NR	NR	NR	NR
Juusela (2020)^33^	2	NP	—	NR	NR	1	1	NR	1	NR	NR	NR	NR	NR
Kalafat (2020)^34^	1	1	0	0	1	NR	NR	0	NR	NR	NR	NR	NR	1; negative
Karami (2020)^35^	1	0	1	0	1	1	1	1	NR	1	NR	NR	NR	NR
Kelly (2020)^36^	1	NP	—	NR	1	NR	1	NR	NR	NR	NR	NR	NR	NR
Lee (2020)^37^	1	1	0	1	0	1	0	NR	NR	NR	NR	NR	NR	NR
Lowe (2020)^38^	1	NR	NR	NR	NR	NR	NR	NR	NR	NR	NR	NR	NR	NR
Schnettler (2020)^39^	1	1	0	1	1	0	1	1	0	0	0	0	0	0
Vlachodimitropoulou Koumoutsea (2020)^40^	2	NR	NR	2	2	2	2	1	2	2	NR	NR	NR	NR
Zamaniyan (2020)^41^	1	1	0	1	1	1	0	NR	NR	NR	NR	NR	1; negative	NR

Only first author is given for each study.

Data are presented as *n* or *n*/*N* (%).

* Including patchy shadowing or ground‐glass opacity.

† Seven cases did not have CT.

‡ Hantoushzadeh (2020)^20^ excluded from calculation because not consecutive case series.

§ Additional information for this study retrieved from Buonsenso (2020)^53^.

ALT, alanine transaminase; AST, aspartate transaminase; CRP, C‐reactive protein; CT, computed tomography; IgG, immunoglobulin‐G; IgM, immunoglobulin‐M; NP, not performed; NR, not reported.

Of the 20 pregnant women with COVID‐19 included in the case reports, two maternal deaths were reported, one each in the case reports of Karami *et al*.^35^ and Zamaniyan *et al*.^41^ from Iran (Table 2). The first case was a 27‐year‐old woman at 30 weeks' gestation who complained of fever, cough and myalgia for 3 days. Her admission laboratory tests showed leukopenia and thrombocytopenia, accompanied by elevated CRP and lactate dehydrogenase levels^35^. Soon after admission, her temperature was noted to be 40°C and her respiratory rate was 55 breaths per min, accompanied by suprasternal and intercostal retraction. Immediate blood tests showed metabolic alkalosis while the patient was under non‐invasive ventilation. She was eventually intubated for mechanical ventilation due to worsening acute respiratory distress syndrome (ARDS), based on clinical and radiological findings. On day 2 of admission, the patient had spontaneous onset of labor and delivered vaginally a cyanotic neonate with no signs of life, that did not respond to neonatal cardiopulmonary resuscitation. One day after birth, the mother developed multiorgan failure (ARDS, acute kidney injury and septic shock) and died^35^. The second case was a 22‐year‐old woman at 32 weeks' gestation who experienced dyspnea, myalgia, anorexia, nausea, non‐productive cough and fever for 4 days^41^. On admission, she was treated with Azithromycin, Ceftriaxone, Kaletra, Tamiflu and hydroxychloroquine. Based on the CT chest finding, lymphopenia, worsening pneumonia symptoms and an unfavorable cervix for induction, a Cesarean delivery was indicated at 33 weeks' gestation. A preterm female infant, weighing 2350 g, was delivered uneventfully, with Apgar scores of 8 and 9 at 1 and 5 min, respectively. The amniotic fluid tested positive for SARS‐CoV‐2 by qRT‐PCR. The immediate post‐delivery nasal and throat swabs of the newborn tested negative for SARS‐CoV‐2, however, repeat testing 24 h later was positive. Such results raised the possibility of vertical transmission. The mother underwent peritoneal dialysis due to ARDS on days 4 and 6 postpartum, and required intubation and mechanical ventilation due to sudden oxygen desaturation to 70% on day 10. She developed emphysema after intubation that resolved spontaneously on day 12; however, her condition deteriorated dramatically and she died on day 15 postpartum^41^.

### Pregnancy and neonatal outcomes

Based on the consecutive case series, the rates of gestational diabetes, hypertensive disorders of pregnancy and pre‐eclampsia did not appear to be higher in pregnant women with COVID‐19 compared to pregnant women without (Table 4). There were only a few cases with hypothyroidism and placenta previa/accreta. A quarter of the cases (72/295) had not been delivered at the time of reporting. Only four cases of spontaneous miscarriage or termination were reported. In the 219 cases that were delivered, including two with twin pregnancy, the majority had a Cesarean section. The gestational age at delivery ranged from 28 to 41 weeks. The Apgar scores at both 1 and 5 min ranged from 7 to 10 (Table 5). Only eight neonates had birth weight < 2500 g. Nearly one‐third of the neonates were transferred to the neonatal intensive care unit (NICU), due mainly to the need for investigation and monitoring as a result of maternal infection. There was one case of neonatal asphyxia and death in the consecutive case series. Of 19 neonates for which laboratory test results were reported, only four and two neonates had increased leukocyte count and CRP, respectively (Table 6). There were no cases of lymphocytopenia and thrombocytopenia. Of note, 29, 29, 155, 19, 19 and 19 cases had nucleic‐acid testing in amniotic fluid, cord blood, neonatal throat swab, neonatal feces, neonatal urine and neonatal gastric juice samples, respectively. All samples, except three neonatal throat‐swab samples from the series of Ferrazzi *et al*.^26^, were negative for SARS‐CoV‐2. In the Iranian case series of nine non‐consecutive cases with severe COVID‐19, there were two cases of intrauterine fetal death (IUFD), that remained undelivered at the time of maternal death (one with twin pregnancy), and two further cases of IUFD amongst the remaining seven cases^20^. An additional two neonatal deaths occurred, as part of a twin pregnancy^20^.

**Table 4:** Pregnancy outcomes of women with COVID‐19 reported in case series and case reports included in systematic review

			Pregnancy complication								Mode of delivery			
Study		*N*	GDM	HPD	PE	HT	PP/PA	Not delivered at time of reporting	Miscarriage/termination	Delivered at time of reporting	CS	COVID‐19 as main CS indication	Vaginal	GA at delivery (weeks)
*Case series*														
Breslin (2020)^19^		43	NR	NR	NR	0	NR	25	0	18	8	0/8	10	NR
Ferrazzi (2020)^26^		42	6	NR	NR	NR	NR	0	0	42	18	10/18	24	NR
Hantoushzadeh (2020)^20^		9‡	NR	NR	NR	NR	NR	2	0	7	6§	2/6	1¶	24–38
Liu (2020)^21^		41	4	3	0	0	NR	25	0	16	16	NR	0	NR
Liu (2020)^22^		13	NR	NR	NR	0	NR	3	0	10	10	5/10	0	NR
Liu (2020)^23^		15	1	0	0	0	1	4	0	11	10	NR	1	NR
Liu (2020)^24^		19	NR	NR	NR	NR	NR	0	0	19	18	NR	1	35 + 2 to 41 + 2
Wu (2020)^25^		23	0	4	0	2	0	0	3	20	18	NR	2	31 + 5 to 40
Yan (2020)^11^		99	7	4	3	0	NR	15	1	83	73	45/73	10	28 + 1 to 41 + 2
Total*		295	18/220 (8.2)	11/178 (6.2)	3/178 (1.7)	2/234 (0.9)	1/38 (2.6)	72/295 (24.4)	4/295 (1.4)	219/295 (74.2)	171/219 (78.1)	60/109 (55.0)	48/219 (21.9)	28–41
													
*Case reports*														
Alonso Díaz (2020)^27^		1	0	1	1	NR	0	0	0	1	1	0	0	38 + 4
Alzamora (2020)^28^		1	NR	0	0	0	0	0	0	1	1	1	0	33 + 3
Buonsenso (2020)^29^, †		2	NR	NR	NR	NR	NR	2	0	2	2	0	0	35 + 7, 38 + 3
Gidlöf (2020)^30^		1	1	1	1	0	0	0	0	1	1	0	0	36 + 2
González Romero (2020)^31^		1	0	0	0	0	0	0	0	1	1	1	0	29
Iqbal (2020)^32^		1	0	0	0	NR	0	0	0	1	0	—	1	39
Juusela (2020)^33^		2	1	1	1	0	0	0	0	2	2	2	0	34 + 1, 39 + 2
Kalafat (2020)^34^		1	NR	0	0	NR	NR	0	0	1	1	1	0	35 + 5
Karami (2020)^35^		1	0	0	0	0	0	0	0	1	0	—	1	30 + 3
Kelly (2020)^36^		1	0	0	0	0	NR	0	0	1	1	0	0	33
Lee (2020)^37^		1	NR	0	0	0	0	0	0	1	1	0	0	37 + 6
Lowe (2020)^38^		1	0	0	0	0	0	0	0	1	0	—	1	40 + 3
Schnettler (2020)^39^		1	0	0	0	0	0	0	0	1	1	1	0	32 + 0
Vlachodimitropoulou Koumoutsea (2020)^40^		2	1	0	0	0	0	0	0	2	2	0	0	36, 35 + 5
Zamaniyan (2020)^41^		1	0	0	0	0	0	0	0	1	1	1	0	32 + 4

Only first author is given for each study.

Data are presented as *n*, *n*/*N*, range or *n*/*N* (%) unless indicated otherwise.

* Hantoushzadeh (2020)^20^ excluded from calculation because not consecutive case series.

† Additional information for this study retrieved from Buonsenso (2020)^53^.

‡ Including two women with intrauterine fetal death (IUFD), who remained undelivered at time of maternal death (one with twin pregnancy).

§ Including one IUFD.

¶ IUFD.

CS, Cesarean section; GA, gestational age; GDM, gestational diabetes mellitus; HPD, hypertensive disorder of pregnancy; HT, hypothyroidism; NR, not reported; PE, pre‐eclampsia; PP/PA, placenta previa/accreta.

**Table 5:** Neonatal outcomes of pregnancies with COVID‐19 reported in case series and case reports included in systematic review

								Neonatal medical complication							
Study		*N* (neonates)	Apgar score (1 min)	Apgar score (5 min)	Birth weight (< 2500 g)	Transferred to NICU	Neonatal asphyxia	Pneumonia	Shortness of breath	Vomiting	Fever	Cough	RT symptoms	Dyspnea	Neonatal mortality
*Case series*															
Breslin (2020)^19^		18	7–10	9–10	NR	3	NR	0	0	0	0	0	1	0	0
Ferrazzi (2020)^26^		42	NR	7–10**	NR	3	NR	NR	NR	NR	NR	NR	1	NR	0
Hantoushzadeh (2020)^20^		8‡	6–9	7–10	5/8	NR	NR	1/4	NR	NR	NR	NR	NR	NR	2§
Liu (2020)^21^		16	NR	NR	NR	NR	0	NR	NR	NR	NR	NR	NR	NR	0
Liu (2020)^22^		10	10¶	NR	NR	1	1	NR	NR	NR	NR	NR	NR	NR	1
Liu (2020)^23^		11	8–10	9–10	NR	NR	0	NR	NR	NR	NR	NR	NR	NR	0
Liu (2020)^24^		19	8¶	9¶	0	0	0	0	0	0	0	0	0	0	0
Wu (2020)^25^		21‡	NR	9–10	NR	NR	0	NR	NR	NR	NR	NR	NR	NR	0
Yan (2020)^11^		84‡	7–10	8–10	8	42	0	NR	NR	NR	NR	NR	NR	NR	0
Total*		221	7–10	7–10	8/103 (7.8)	49/173 (28.3)	1/161 (0.6)	0/37 (0)	0/37 (0)	0/37 (0)	0/37 (0)	0/37 (0)	2/79 (2.5)	0/37 (0)	1/221 (0.5)
*Case reports*															
Alonso Díaz (2020)^27^		1	7	9	0	0	0	1	1	0	0	0	1	1	0
Alzamora (2020)^28^		1	6	8	0	1	0	0	0	NR	0	1	1	1	0
Buonsenso (2020)^29^, †		2	8, 9	9, 10	1	0	0	0	0	0	0	0	0	0	0
Gidlöf (2020)^30^		2§	9	10	1	0	0	0	0	1	0	0	1	0	0
González Romero (2020)^31^		1	8	10	1	1	0	0	0	0	0	0	0	0	0
Iqbal (2020)^32^		1	8	9	NR	0	0	0	NR	NR	NR	NR	NR	NR	0
Juusela (2020)^33^		2	NR	NR	NR	NR	NR	NR	NR	NR	NR	NR	NR	NR	0
Kalafat (2020)^34^		1	NR	9	0	0	0	0	NR	NR	NR	NR	NR	NR	0
Karami (2020)^35^		1	0	0	NR	NR	NR	NR	NR	NR	NR	NR	NR	NR	1
Kelly (2020)^36^		1	NR	1	NR	1	0	0	0	0	0	0	0	0	0
Lee (2020)^37^		1	9	10	0	0	0	0	NR	NR	NR	NR	NR	NR	0
Lowe (2020)^38^		1	9	9	NR	0	0	0	0	0	0	0	0	0	0
Schnettler (2020)^39^		1	NR	NR	NR	1	0	0	0	0	0	0	0	0	0
Vlachodimitropoulou Koumoutsea (2020)^40^		2	9, 4	9	0	0	0	NR	NR	NR	NR	NR	NR	NR	0
Zamaniyan (2020)^41^		1	8	9	1	0	0	0	0	0	1	0	0	0	0

Only first author is given for each study.

Data are presented as *n*, range or *n*/*N* (%) unless indicated otherwise.

* Hantoushzadeh (2020)^20^ excluded from calculation because not consecutive case series.

† Additional information for this study retrieved from Buonsenso (2020)^53^.

‡ Including one twin pregnancy.

§ Twin pregnancy.

¶ Mean value.

** Two very preterm newborns had 5‐min Apgar score < 7.

NICU, neonatal intensive care unit; NR, not reported; RT, respiratory tract.

**Table 6:** Laboratory findings and biological sample testing in neonates born to mothers with COVID‐19 reported in case series and case reports included in systematic review

			Laboratory						Biological sample						
Study		*N* (neonates)	Leukocyte (high)	Lymphocyte (low)	Platelet (low)	CRP (high)	IgG (high)	IgM (high)	Amniotic fluid	Cord blood	Placenta	Throat swab	Feces	Urine	Gastric juice
*Case series*															
Breslin (2020)^19^		18	NR	NR	NR	NR	NR	NR	NR	NR	NR	18; all negative	NR	NR	NR
Ferrazzi (2020)^26^		42	NR	NR	NR	NR	NR	NR	NR	NR	NR	42; 39 negative, 3 positive¶	NR	NR	NR
Hantoushzadeh (2020)^20^		7	NR	1/4	NR	NR	NR	NR	NR	NR	NR	5; all negative	NR	NR	NR
Liu (2020)^21^		16	NR	NR	NR	NR	NR	NR	NR	NR	NR	NR	NR	NR	NR
Liu (2020)^22^		10	NR	NR	NR	NR	NR	NR	NR	NR	NR	NR	NR	NR	NR
Liu (2020)^23^		11	NR	NR	NR	NR	NR	NR	NR	NR	NR	NR	NR	NR	NR
Liu (2020)^24^		19	4	0	0	2	NR	NR	19; all negative	19; all negative	NR	19; all negative	19; all negative	19; all negative	19; all negative
Wu (2020)^25^		21‡	NR	NR	NR	NR	NR	NR	NR	NR	NR	4; all negative	NR	NR	NR
Yan (2020)^11^		84‡	NR	NR	NR	NR	NR	NR	10; all negative	10; all negative	NR	72; all negative	NR	NR	NR
Total*		221	4/19 (21.1)	0/19 (0)	0/19 (0)	2/19 (10.5)	NR	NR	—	—	—	—	—	—	—
*Case reports*															
Alonso Díaz (2020)^27^		1	NR	NR	NR	0	NR	NR	NR	NR	NR	1; negative	NR	NR	NR
Alzamora (2020)^28^		1	0	0	0	0	0	0	NR	NR	NR	1; positive	NR	NR	NR
Buonsenso (2020)^29^, †		2	NR	NR	NR	NR	1; positive	NR	NR	2; negative	2; 1 negative, 1 positive	2; negative	NT	NR	NR
Gidlöf (2020)^30^		2§	NR	NR	NR	NR	NR	NR	NR	NR	NR	2; negative	NR	NR	NR
González Romero (2020)^31^		1	NR	NR	NR	NR	NR	NR	NR	NR	NR	1; negative	NR	NR	NR
Iqbal (2020)^32^		1	NR	NR	NR	NR	NR	NR	NR	NR	NR	1; negative	NR	NR	NR
Juusela (2020)^33^		2	NR	NR	NR	NR	NR	NR	NR	NR	NR	NR	NR	NR	NR
Kalafat (2020)^34^		1	NR	NR	NR	NR	NR	NR	NR	1; negative	1; negative	1; negative	NR	NR	NR
Karami (2020)^35^		1	NR	NR	NR	NR	NR	NR	NR	NR	NR	NR	NR	NR	NR
Kelly (2020)^36^		1	NR	NR	NR	NR	NR	NR	NR	NR	NR	1; negative	NR	NR	NR
Lee (2020)^37^		1	NR	NR	NR	NR	NR	NR	1; negative	1; negative	1; negative	1; negative	NR	NR	NR
Lowe (2020)^38^		1	NR	NR	NR	NR	NR	NR	NR	NR	NR	1; negative	NR	NR	NR
Schnettler (2020)^39^		1	NR	NR	NR	NR	NR	NR	1; negative	NR	NR	1; negative	NR	NR	NR
Vlachodimitropoulou Koumoutsea (2020)^40^		2	NR	NR	NR	NR	NR	NR	NR	NR	NR	NR	NR	NR	NR
Zamaniyan (2020)^41^		1	NR	NR	NR	NR	NR	NR	1; positive	1; neg	NR	1; neg**	NR	NR	NR

Only first author is given for each study.

Data are presented as *n* or *n*/*N* (%) unless indicated otherwise.

* Hantoushzadeh (2020)^20^ excluded from calculation because not consecutive case series.

† Additional information for this study retrieved from Buonsenso (2020)^53^.

‡ Including one twin pregnancy.

§ Twin pregnancy.

¶ Testing at day 1 and day 3 or 4 postpartum.

** 24 h after birth, throat swab became positive.

CRP, C‐reactive protein; IgG, immunoglobulin‐G; IgM, immunoglobulin‐M; NR, not reported.

In one case report, the neonatal throat swab tested positive for SARS‐CoV‐2 (Table 6)^28^. The mother was a 41‐year‐old woman with pre‐existing diabetes mellitus and significant COVID‐19 exposure from immediate family members. She presented at 33 weeks' gestation with a 4‐day history of malaise, fatigue, low‐grade fever and progressive shortness of breath. The nasopharyngeal swab of the patient was positive for SARS‐CoV‐2. The patient developed severe respiratory failure requiring mechanical ventilation on day 5 of disease onset. She was started on azithromycin, hydroxychloroquine, meropenem, vancomycin and oseltamivir. The patient underwent a preterm Cesarean delivery due to compromised respiratory status. Neonatal isolation was implemented immediately after birth, without delayed cord clamping or skin‐to‐skin contact. The neonate weighed 2970 g, with Apgar scores of 6 and 8 at 1 and 5 min, respectively. The neonate was not exposed to family members. Breastfeeding was not initiated. The neonate was placed in the NICU with no other COVID‐19 cases, as this was the first pediatric case at the institution. Chest X‐ray showed no abnormalities. At 16 h after delivery, the neonatal nasopharyngeal swab tested positive for SARS‐CoV‐2 by qRT‐PCR, which was repeated 48 h later and remained positive. However, anti‐SARS‐CoV‐2 immunoglobulin‐M (IgM) and ‐G (IgG) were negative at birth. The possibility of postpartum neonatal infection cannot be completely excluded because of the delay in testing. The newborn required ventilatory support for 12 h, after which he was extubated and placed on continuous positive airway pressure, with favorable outcome.

### Risk of bias

All case series, except one^20^, had low risk of bias due to clear inclusion criteria, proper evaluation of the disease status, consecutive and complete enrolment, clarity of the reported variables and appropriate statistical analysis. Additionally, although most of the case reports failed in the reporting of other adverse or unanticipated events besides the main case presentation, all presented the clinical case clearly and provided takeaway lessons.

## DISCUSSION

### Main findings

This systematic review has demonstrated that, first, the most common reported symptoms of COVID‐19 are fever, cough, dyspnea/shortness of breath, fatigue and myalgia; second, on admission, most cases have patchy shadowing or ground‐glass opacity on CT of the chest, while normal or low leukocyte count, lymphocytopenia and raised CRP were the most common laboratory findings observed in pregnant patients with COVID‐19; third, the rate of severe pneumonia reported amongst the case series ranged from 0% to 14%; fourth, of the 324 pregnant women included, seven maternal deaths were reported in a case series of nine non‐consecutive cases with severe COVID‐19 and two amongst the case reports (as of 28 April 2020); fifth, COVID‐19 does not appear to increase the risk of adverse pregnancy outcome, such as pre‐eclampsia; sixth, only a few cases of spontaneous miscarriage or termination have been reported in pregnancies with COVID‐19; seventh, there is a lack of completeness of pregnancy outcome data; eighth, based on women who had delivered at the time of reporting, the gestational age at delivery ranged from 28 to 41 weeks and the majority of cases had Cesarean delivery; and ninth, among the consecutive case series, there were three reported cases of neonates testing positive for SARS‐CoV‐2 and among the case reports, there was one case each with positive SARS‐CoV‐2 in amniotic fluid and neonatal throat swab.

### Comparison with previous studies

At present, there is much controversy relating to the possibility of vertical mother‐to‐baby transmission of SARS‐CoV‐2. In two earlier studies, with a combined total of 10 pregnant women with COVID‐19 in the third trimester, amniotic‐fluid, cord‐blood and neonatal throat‐swab samples tested negative for SARS‐CoV‐2, suggesting there was no evidence of vertical transmission in women who developed COVID‐19 pneumonia in late pregnancy^10^, ^43^. In another series, a neonate born to a pregnant woman with COVID‐19 tested positive for SARS‐CoV‐2 in the pharyngeal swab sample 36 h after birth, but it was subsequently confirmed that qRT‐PCR testing of the placenta and cord blood was negative for SARS‐CoV‐2, suggesting that intrauterine vertical transmission might not have occurred^44^, ^45^.

Two research letters^12^, ^13^ have suggested the possibility of vertical transmission of SARS‐CoV‐2, based on the presence of IgM antibodies in blood drawn from three neonates born to mothers with COVID‐19. However, for all three, the respiratory samples tested negative for SARS‐CoV‐2. Furthermore, one neonate had repeat testing for SARS‐CoV‐2 IgG and IgM antibodies, and the observed rapid decline of IgG levels within 14 days, along with a decline in IgM antibodies, strongly suggested that neonatal SARS‐CoV‐2 IgG antibodies were derived transplacentally from the mother and not actively induced by the presumed neonatal infection.

The findings of the case of maternal death reported by Zamaniyan *et al*.^41^, in which amniotic fluid and neonatal nasal and throat swabs tested positive for SARS‐CoV‐2, and the case report by Alzamora *et al*.^28^, in which the neonatal throat swab tested positive for SARS‐CoV‐2, have again raised the possibility of vertical transmission. The authors of the former case report could not measure specific antibodies in the neonate, which might have provided supplementary evidence for vertical transmission of SARS‐CoV‐2. This case is of particular interest because the pregnant patient might have had virulent COVID‐19, which was not immediately apparent prior to delivery, as her clinical course deteriorated dramatically only after delivery. The common features of these two cases of neonatal SARS‐CoV‐2 infection are that the pregnant patients contracted the virus preterm and had severe COVID‐19. There appear to be at least two ways through which SARS‐CoV‐2 can cause intrauterine infection by vertical transmission. Angiotensin‐converting enzyme 2 (ACE2), which has been indicated recently as the putative surface receptor of sensitive cells for SARS‐CoV‐2^46^, has been shown to be expressed in human placentas^47^. This opens up the possibility that SARS‐CoV‐2 could spread transplacentally through ACE2. Specifically, the viral surface spike glycoprotein (S‐protein) of SARS‐CoV‐2 is cleaved by transmembrane protease serine 2 (TMPRSS2) to facilitate efficiency of entry and viral replication, and there is emerging evidence that SARS‐CoV‐2 co‐opts and recruits additional host proteases for transmissibility^48^, ^49^. It is, however, unknown whether there is placental expression of host proteases (such as TMPRSS2 and others) necessary for cleavage of the S‐protein and receptor priming. On the other hand, placental barrier damage caused by severe maternal hypoxemia in women with COVID‐19 could also be one potential way through which SARS‐CoV‐2 can cause intrauterine infection. There is an urgent need to investigate further the possibility of vertical transmission^50^ of SARS‐CoV‐2.

The second case of maternal death, that was reported by Karami *et al*.^35^, underwent autopsy and histopathologic evaluation of paraffin‐embedded lung tissue that showed alveolar spaces with focal hyaline membrane, pneumocyte proliferation and metaplastic changes. There was evidence of viral pneumonia (viral cytopathic effect, including multinucleation and nuclear atypia and a mild increase in alveolar wall thickness). These findings are comparable to observations in non‐pregnant cases^51^. The Iranian case series reported the maternal death of seven pregnant women presenting with severe COVID‐19, during the latter second or third trimester, over a 30‐day interval^20^. All cases received a three‐drug regimen, which included oseltamivir 75 mg PO, every 12 h for 5 days, hydroxychloroquine sulfate 400 mg PO daily or chloroquine sulfate 1000 mg tablet PO, as a single dose, and lopinavir/ritonavir 400/100 mg PO, every 12 h for 5 days. There were two key features in these cases. First, the average maternal age in this series was higher than that in others (36.7 ± 7.3 *vs* 30.3 ± 3.6 years)^20^. Second, according to the authors, none of the pregnant patients had pre‐existing comorbidities, such as hypertension, cardiovascular disease and asthma^20^. The authors believed that the COVID‐19 pregnant patients did not receive suboptimal care and raised concern regarding the potential for maternal death among pregnant women diagnosed with COVID‐19 in the latter trimester. Notably, the authors did not provide details of the cause(s) of death^20^.

### Strengths and limitations

This systematic review has several strengths. We undertook a meticulous process in identifying duplicate reporting: (i) case reports from China were excluded; (ii) when a hospital had published their cases more than once, only the paper with the most data or published on the later date was included; (iii) we were able to identify the duplicates from our own publication^11^, which is the biggest cohort series with available pregnancy outcome data from China; and (iv) when necessary, we contacted the corresponding authors of included studies using their native language, i.e. Chinese and Spanish, thus increasing the chance of receiving a response from them. Moreover, we did not apply any language restrictions in order to capture a global picture of the impact of COVID‐19 on pregnancy and perinatal outcomes, and most importantly, we had the capability to ensure that data from China could be accurately interpreted and recorded.

This study also has several major limitations. The main limitation arises from the nature of the included studies. Although case reports are an important source of knowledge, they are not suitable for inference statistics and are expected to suffer from high publication bias. On the other hand, the main limitation of case series is the lack of internal controls; thus, they typically yield very low‐quality evidence. However, at this point in time when higher‐quality studies on COVID‐19 in pregnancy are yet to be conducted, both case reports and case series provide valuable and necessary information to guide clinicians in their decision making. We included papers that reported at least one maternal, perinatal or neonatal outcome. Given the severity of the pandemic and the urgent need for knowledge sharing, we felt that it would still be valuable to include papers that had provided limited pregnancy outcome data. In view of the low rate of miscarriage, it is likely that asymptomatic cases with SARS‐CoV‐2 infection resulting in a miscarriage were not identified and, therefore, are under‐reported. In our opinion, it was simply a failure of appropriate diagnosis and there was no reporting/publication bias. We were unable to include the second largest cohort series with available pregnancy outcome data from China because the data were pooled from a national registry^52^. Despite contacting the corresponding author directly, it was not possible to identify the actual source of cases. All 68 pregnant patients with pregnancy outcome data were cases from Wuhan and the authors did not make reference to previously published cases. In our publication^11^, we included 77 cases from Wuhan and we had strong reasons to believe that there was significant overlap between the two series; therefore, we regrettably had to exclude the data from Chen *et al*.^52^ in order to avoid inflating the number of SARS‐CoV‐2‐infected pregnant cases from China. We also learned that pregnant women with COVID‐19 could have been transferred to other hospitals, which made it difficult to determine duplicate reporting as cases could have been reported by both hospitals in which the woman received care. We decided to combine laboratory‐confirmed and clinically diagnosed COVID‐19 cases in our analysis because both groups had similar outcomes^11^. We believed it was important to include the clinically diagnosed cases in this review, as such cases had typical COVID‐19 chest CT findings and significant epidemiological exposure. As of 28 April 2020, maternal death had been reported in seven and two cases in the non‐consecutive case series^20^ and amongst the case reports^35^, ^41^, respectively. Traditionally, any maternal death requires an extensive process of investigation and therefore it is not immediately reported and made public. It was not possible to determine COVID‐19‐related maternal case fatality rate and such data will come to light only at the end of the pandemic.

### Conclusions

Despite the increasing number of published studies on COVID‐19 in pregnancy, there are insufficient good‐quality data to draw unbiased conclusions with regard to the severity of COVID‐19 or specific complications of the disease in pregnant women, as well as vertical transmission and perinatal and neonatal complications. In order to answer specific questions in relation to the impact of COVID‐19 on pregnant women and their fetuses through meaningful good‐quality research, we urge researchers and investigators to present complete outcome data and reference previously published cases in their publications, and to record such reporting when the data of a case are entered into a registry or several registries.

## Supporting information


**Appendix S1** Systematic literature search
**Table S1** Details of contact with corresponding authors when further clarification of data was needed
**Table S2** Excluded papers and reason(s) for exclusion
**Table S3** Quality assessment of case series included in systematic review
**Table S4** Quality assessment of case reports included in systematic review
