# Supplementary material for: Effect of coronavirus disease 2019 (COVID‐19) on maternal, perinatal and neonatal outcome: systematic review
Source: Ultrasound Obstet Gynecol. 2020 Jul 1;56(1):15–27. doi: 10.1002/uog.22088 (PMC7276742; doi:10.1002/uog.22088)
Supplement: Supplementary file 1 — Appendix S1 Systematic literature search Table S1 Details of contact with corresponding authors when further clarification of data was needed Table S2 Excluded papers and reason(s) for exclusion Table S3 Quality assessment of case series included in systematic review Table S4 Quality assessment of case reports included in systematic review [file UOG-56-15-s001.docx]

**Appendix S1** Systematic search

(COVID-19[tw] OR COVID19[tw] OR 2019-nCoV[tw] OR nCoV-2019[tw] OR coronavirus 2019[tw] OR 2019 coronavirus[tw] OR corona virus* 2019[tw] OR 2019 corona virus*[tw] OR novel coronavirus[tw] OR novel corona virus*[tw] OR “severe acute respiratory syndrome coronavirus 2”[tw] OR coronavirus disease-19[tw] OR coronavirus disease-2019[tw] OR coronavirus disease 2019[tw] OR corona virus disease 2019[tw] OR corona virus disease-2019[tw] OR corona virus disease-19[tw] OR new coronavirus*[tw] OR new corona virus*[tw] OR SARS-CoV-2[tw] OR SARS-coronavirus 2[tw] OR “coronavirus 2”[tw] OR “spike glycoprotein, COVID-19 virus” [Supplementary Concept] OR “LAMP assay” [Supplementary Concept] OR “COVID-19 serotherapy” [Supplementary Concept] OR “COVID-19 drug treatment” [Supplementary Concept] OR “COVID-19 diagnostic testing” [Supplementary Concept] OR “COVID-19 vaccine” [Supplementary Concept] OR “severe acute respiratory syndrome coronavirus 2” [Supplementary Concept] OR (Wuhan[tiab] AND (coronavirus*[tiab] OR corona virus*[tiab])) AND (2019:2020[pdat])) AND (((((“Infant”[Mesh] OR infan*[tw] OR “Infant, Newborn”[Mesh] OR newborn*[tw] OR “Premature Birth”[tw] OR premature births[tw] OR “Infant, Premature”[Mesh] OR premature infant*[tw] OR premature bab*[tw] OR premature child*[tw] OR neonatal prematurity[tw] OR preterm birth*[tw] OR preterm infant*[tw] OR preterm bab*[tw] OR preterm child*[tw] OR pre-term birth*[tw] OR pre-term infant*[tw] OR pre-term bab*[tw] OR pre-term child*[tw] OR “Infant, Extremely Premature”[Mesh] OR extremely premature[tw] OR extremely preterm[tw] OR extremely pre-term[tw] OR “Infant, Low Birth Weight”[Mesh] OR low birth weight*[tw] OR “Infant, Very Low Birth Weight”[Mesh] OR very low birth weight*[tw] OR “Infant, Small for Gestational Age”[Mesh] OR “Infant, Postmature”[Mesh] OR postmature bab*[tw] OR postmature infant*[tw] OR postmature child*[tw] OR Perinatology[tw] OR perinatal*[tw] OR antepartum[tw] OR ante-partum[tw] OR intra-partum[tw] OR intrapartum[tw] OR Neonatology[tw] OR neonat*[tw] OR neo-nat*[tw] OR postnatal*[tw] OR post-natal*[tw] OR fetus*[tw] OR foetus*[tw] OR fetal*[tw] OR foetal*[tw] OR baby*[tw] OR babies[tw])) OR (maternal and perinatal outcomes with COVID-19 a systematic review)) OR (Pregnancy[tw] OR pregnancies[tw] OR pregnan*[tw] OR gestation*[tw] OR delivery[tw] OR deliveries[tw])))

This search is continuously updated with new terms and conditions and freely available at: <https://bvcsmaquedano.wordpress.com/2020/04/17/covid-19-pregnancy/>

**Table S1** Details of contact with corresponding authors when further clarification of data was needed

| **Study** | **Country** | **N** | **Email** | **Reply** | **Respondence** |
| --- | --- | --- | --- | --- | --- |
| Gonzalez Romero (2020)^31^ | Spain | 1 | 1. Could you confirm that the neonate was tested for SARS-CoV-2? 2. Would it be possible to complete neonatal outcome and confirm if there were any pregnancy complications? | Yes | 1. Yes, two throat swabs were negative: immediately after birth and 48 hours later. 2. Gestational age, Apgar scores, birthweight, cord pH and follow up on day 28 after birth were provided. No pregnancy complications reported. |
| Liu (2020)^24^ | China | 19 | 1. As far as you know, whether the mothers of the 19 neonates included in your papers have been published in other papers? 2. As you reported in your paper, the cases came from two hospitals: Union Hospital and Tongji Hospital. How many neonates came from these two hospitals separately? 3. Any idea whether the mothers diagnosed and delivered at the same hospital, or they were transferred to other hospitals? | Yes | 1. Seven of the 19 patients have been reported by another obstetrician from Tongji Hospital. 2. Twelve patients were from Tongji Hospital and seven patients were from Union Hospital West, which is a different hospital from Union Hospital, Wuhan. 3. All 19 pregnant women were diagnosed and delivered in Tongji Hospital or Union Hospital West. |
| Wu (2020)^25^ | China | 23 | 1. Do you know whether the 23 pregnant women included in your paper have been published in other papers? 2. Were these 23 pregnant women diagnosed and delivered at your hospital? Could they have been transferred to other hospitals? | Yes | 1. The 23 pregnant women of our study have not been published in our articles so far. But other clinicians of our department are preparing a manuscript of these pregnant women regarding other aspects. 2. Some of the pregnant women were diagnosed in our hospital, the others were diagnosed by other hospitals and were then transferred to our hospital. All these 23 patients were treated and delivered in our hospital as our hospital is a designated hospital for pregnant women with COVID-19 in China. |
| Liu (2020)^21^ | China | 41 | 1. Do you know whether the 41 pregnant women included in your paper have been published in other papers? 2. Were these 41 pregnant women diagnosed and delivered at your hospital? Could they have been transferred to other hospitals? | Yes | The 41 pregnant women with COVID-19 in our study might have also been published or being published in other articles by other clinicians of our hospital. |
| Liu (2020)^23^ | China | 15 | 1. Do you know whether the 15 pregnant women included in your paper have been published in other papers? 2. Were these 15 pregnant women diagnosed and delivered at your hospital? Could they have been transferred to other hospitals? | No |  |
| Zhu (2020)^a^ | China | 9 | 1. Do you know whether the mothers of the 10 neonates included in your paper have been published in other papers? 2. As reported in your paper, the cases came from 5 hospitals in Hubei. Would you be able to tell us which of these 5 hospitals were and how many case(s) came from each hospital separately? 3. Do you know whether the mothers were diagnosed and delivered at the same hospital, or they were transferred to other hospitals? | No |  |
| Zeng (2020)^b^ | China | 33 | 1. Do you know whether the mothers of the 33 neonates included in your paper have been published in other papers? 2. As reported in your paper, the 33 neonates were born to mothers with COVID-19 in Wuhan. Would you be able to tell us which of these hospitals were and how many case(s) came from each hospital separately? 3. Do you know whether the mothers were diagnosed and delivered at the same hospitals, or they were transferred to other hospitals? | No |  |

**Table S2** Excluded papers and reason(s) for exclusion

| **Study** | **Country** | **N** | **Study Design** | **Exclusion reason** |
| --- | --- | --- | --- | --- |
| Chen (2020)^c^ | China | 5 | Case series | 1. Likely included in Liu (2020)^21^ 2. Case series from China that included less than 10 cases |
| Chen (2020)^d^ | China | 3 | Retrospective study | 1. Likely included in Liu (2020)^23^ 2. Case series from China that included less than 10 cases |
| Chen (2020)^10^ | China | 9 | Retrospective study | 1. Included in Yan (2020)^11^ 2. Case series from China that included less than 10 cases |
| Chen (2020)^52^ | China | 118 | Case series | Likely included in Yan (2020)^11^ |
| Chen (2020)^e^ | China | 17 | Case series | Likely included in Yan (2020)^11^ |
| Chen (2020)^f^ | China | 4 | Case series | 1. Case series from China that included less than 10 cases 2. Mainly focused on neonates 3. Likely included in Yan (2020)^11^ |
| Dong (2020)^13^ | China | 1 | Case report | Case report from China |
| Fan (2020)^g^ | China | 2 | Case series | 1. Case series from China that included less than 10 cases 2. Likely included in Yan (2020)^11^ |
| He (2020)^h^ | China | 1 | Case report | Case report from China |
| Kang (2020)^i^ | China | 1 | Case report | Case report from China |
| Khan (2020)^j^ | China | 17 | Case series | Likely included in Yan (2020)^11^ |
| Khan (2020)^k^ | China | 3 | Case report | Likely included in Yan (2020)^11^ |
| Lei (2020)^43^ | China | 9 | Retrospective study | 1. Included in Yan (2020)^11^ 2. Case series from China that included less than 10 cases |
| Li (2020)^l^ | China | 1 | Case report | Case report from China |
| Li (2020)^m^ | China | 1 | Case report | Case report from China |
| Li (2020)^n^ | China | 34 | Case-control study | Likely included in Liu (2020)^21^ |
| Li (2020)^o^ | China | 12 | Retrospective study | Likely included in Yan (2020)^11^ |
| Liao (2020)^p^ | China | 1 | Case report | Case report from China |
| Liu (2020)^q^ | China | 3 | Case series | 1. Case series from China that included less than 10 cases 2. Likely included in Liu (2020)^24^ |
| Wang (2020)^r^ | China | 1 | Case report | Case report from China |
| Wang (2020)^45^ | China | 1 | Case report | Case report from China |
| Wang (2020)^s^ | China | 1 | Case report | Case report from China |
| Wen (2020)^t^ | China | 1 | Case report | Case report from China |
| Xiong (2020)^u^ | China | 1 | Case report | 1. Included in Yan (2020)^11^ 2. Case report from China |
| Yao (2020)^v^ | China | 1 | Case report | Case report from China |
| Yu (2020)^44^ | China | 7 | Retrospective study | 1. Included in Liu (2020)^24^ 2. Case series from China that included less than 10 cases |
| Zambrano (2020)^w^ | Central America | 1 | Case report | 1. Missing outcome data |
| Zeng (2020)^b^ | China | 33 | Cohort study | 1. Likely included in Yan (2020)^11^ 2. Mainly focused on neonates |
| Zeng (2020)^12^ | China | 6 | Retrospective study | 1. Included in Yan (2020)^11^ 2. Case series from China that included less than 10 cases |
| Zhang (2020)^x^ | China | 4 | Case series | 1. Likely included in Yan (2020)^11^ 2. Mainly focused on neonates 3. Case series from China that included less than 10 cases |
| Zhang (2020)^y^ | China | 16 | Retrospective study | Included in Yan (2020)^11^ |
| Zhao (2020)^z^ | China | 1 | Case report | Case report from China |
| Zhao (2020)^aa^ | China | 1 | Case report | Case report from China |
| Zhou (2020)^bb^ | China | 1 | Case report | Case report from China |
| Zhu (2020)^a^ | China | 9 | Case series | 1. Mainly focused on neonates 2. Likely included in Yan (2020)^11^ 3. Case series from China that included less than 10 cases |
| Zhuang (2020)^cc^ | China | 1 | Case report | Case report from China |

**References**:

1. Zhu H, Wang L, Fang C, Peng S, Zhang L, Chang G, Xia S, Zhou W. Clinical analysis of 10 neonates born to mothers with 2019-nCoV pneumonia. *Transl Pediatr*. 2020;**9**(1):51-60. doi: 10.21037/tp.2020.02.06.
2. Zeng L, Xia S, Yuan W, Yan K, Xiao F, Shao J, Zhou W. Neonatal Early-Onset Infection With SARS-CoV-2 in 33 Neonates Born to Mothers With COVID-19 in Wuhan, China. *JAMA Pediatr*. 2020:e200878. doi: 10.1001/jamapediatrics.2020.0878.
3. Chen S, Liao E, Cao D, Gao Y, Sun G, Shao Y. Clinical analysis of pregnant women with 2019 novel coronavirus pneumonia. *J Med Virol*. 2020. doi: 10.1002/jmv.25789.
4. Chen S, Huang B, Luo DJ, Li X, Yang F, Zhao Y, Nie X, Huang BX. [Pregnant women with new coronavirus infection: a clinical characteristics and placental pathological analysis of three cases]. *Zhonghua Bing Li Xue Za Zhi*. 2020;**49**(0):E005. Chinese. doi: 10.3760/cma.j.cn112151-20200225-00138.
5. Chen R, Zhang Y, Huang L, Cheng BH, Xia ZY, Meng QT. Safety and efficacy of different anesthetic regimens for parturients with COVID-19 undergoing Cesarean delivery: a case series of 17 patients. *Can J Anaesth*. 2020:1–9. doi: 10.1007/s12630-020-01630-7.
6. Chen Y, Peng H, Wang L, Zhao Y, Zeng L, Gao H, Liu Y. Infants Born to Mothers With a New Coronavirus (COVID-19). *Front Pediatr*. 2020;**8**:104. doi: 10.3389/fped.2020.00104.
7. Fan C, Lei D, Fang C, Li C, Wang M, Liu Y, Bao Y, Sun Y, Huang J, Guo Y, Yu Y, Wang S. Perinatal Transmission of COVID-19 Associated SARS-CoV-2: Should We Worry? *Clin Infect Dis*. 2020:ciaa226. doi: 10.1093/cid/ciaa226.
8. He S, Wang D, Chi R, Ding D, Yu Y, He M, Li W, Chi C, Shi M. Death of a neonate born to a critically ill mother with COVID-19: a case report. *Chin J Perinat Med*. 2020;**23**(4):217-220. Chinese.
9. Kang X, Zhang R, He H, Yao Y, Zheng Y, Wen X, Zhu S. [Anesthesia management in cesarean section for a patient with coronavirus disease 2019]. *Zhejiang Da Xue Xue Bao Yi Xue Ban.* 2020;**49**(1). Chinese.
10. Khan S, Jun L, Nawsherwan, Siddique R, Li Y, Han G, Xue M, Nabi G, Liu J. Association of COVID-19 with pregnancy outcomes in health-care workers and general women. *Clin Microbiol Infect.* 2020:S1198-743X(20)30180-4. doi: 10.1016/j.cmi.2020.03.034.
11. Khan S, Peng L, Siddique R, Nabi G, Nawsherwan, Xue M, Liu J, Han G. Impact of COVID-19 infection on pregnancy outcomes and the risk of maternal-to-neonatal intrapartum transmission of COVID-19 during natural birth. *Infect Control Hosp Epidemiol*. 2020:1-3. doi: 10.1017/ice.2020.84.
12. Li Y, Zhao R, Zheng S, Chen X, Wang J, Sheng X, Zhou J, Cai H, Fang Q, Yu F, Fan J, Xu K, Chen Y, Sheng J. Lack of Vertical Transmission of Severe Acute Respiratory Syndrome Coronavirus 2, China. *Emerg Infect Dis*. 2020;**26**(6). doi: 10.3201/eid2606.200287.
13. Li M, Xu M, Zham W, Han T, Zhang G, Lu Y. 2019新型冠状病毒母婴感染一例.*Chin J Infect Dis*. 2020;**38**(2):117-118. Chinese
14. Li N, Han L, Peng M, Lv Y, Ouyang Y, Liu K, Yue L, Li Q, Sun G, Chen L, Yang L. Maternal and neonatal outcomes of pregnant women with COVID-19 pneumonia: a case-control study. *Clin Infect Dis*. 2020:ciaa352. doi: 10.1093/cid/ciaa352.
15. Li L, Wang L, Zeng F, Liu F, Peng Z, Xie B, Liu C, Zha Y. The application value of low-dose CT scan in pregnant women with corona virus disease 2019. doi: 10.3760/cma.j.issn.0254-5098.2020.05.000. Chinese
16. Liao X, Yang H, Kong J, Yang H. Chest CT Findings in a Pregnant Patient with 2019 Novel Coronavirus Disease. *Balkan Med J*. 2020. doi: 10.4274/balkanmedj.galenos.2020.2020.3.89.
17. Liu W, Wang Q, Zhang Q, Chen L, Chen J, Zhang B, Lu Y, Wang S, Xia L, Huang L, Wang K, Liang L, Zhang Y, Turtle L, Lissauer D, Lan K, Feng L, Yu H, Liu Y, Sun Z. Coronavirus disease 2019 (COVID-19) during pregnancy: a case series. 2020. www. preprint.org.
18. Wang J, Wang D, Chen GC, Tao XW, Zeng LK. [SARS-CoV-2 infection with gastrointestinal symptoms as the first manifestation in a neonate]. *Zhongguo Dang Dai Er Ke Za Zhi.* 2020;**22**(3):211-214. Chinese.
19. Wang X, Zhou Z, Zhang J, Zhu F, Tang Y, Shen X. A case of 2019 Novel Coronavirus in a pregnant woman with preterm delivery. *Clin Infect Dis*. 2020:ciaa200. doi: 10.1093/cid/ciaa200.
20. Wen R, Sun Y, Xing QS. A patient with SARS-CoV-2 infection during pregnancy in Qingdao, China. *J Microbiol Immunol Infect*. 2020. doi: 10.1016/j.jmii.2020.03.004.
21. Xiong X, Wei H, Zhang Z, Chang J, Ma X, Gao X, Chen Q, Pang Q. Vaginal delivery report of a healthy neonate born to a convalescent mother with COVID--19. *J Med Virol*. 2020. doi: 10.1002/jmv.25857.
22. Yao L, Wang J, Zhao J,Cui J, Hu Z. Asymptomatic COVID-19 infection in pregnant women in the third trimester: a case report. Chin J Perinat Med. 2020;23(3):229-231. Chinese
23. Zambrano LI, Fuentes-Barahona IC, Bejarano-Torres DA, Bustillo C, Gonzales G, Vallecillo-Chinchilla G, Sanchez-Martínez FE, Valle-Reconco JA, Sierra M, Bonilla-Aldana DK, Cardona-Ospina JA, Rodríguez-Morales AJ. A pregnant woman with COVID-19 in Central America. Travel Med Infect Dis. 2020:101639. doi: 10.1016/j.tmaid.2020.101639.
24. Zhang ZJ, Yu XJ, Fu T, Liu Y, Jiang Y, Yang BX, Bi Y. Novel Coronavirus Infection in Newborn Babies Under 28 Days in China. *Eur Respir J*. 2020:2000697. doi: 10.1183/13993003.00697-2020.
25. Zhang L, Jiang Y, Wei M, Cheng BH, Zhou XC, Li J, Tian JH, Dong L, Hu RH. [Analysis of the pregnancy outcomes in pregnant women with COVID-19 in Hubei Province]. *Zhonghua Fu Chan Ke Za Zhi*. 2020;**55**(0):E009. Chinese. doi: 10.3760/cma.j.cn112141-20200218-00111. Chinese
26. Zhao R, Wang H, Xu K, Sheng J. 妊娠合并新型冠状病毒肺炎1例.*Zhe Jiang Yi Xue*. 2020;**42**(4):303-305.Chinese
27. Zhao Y, Zou L, Dong M, Liu X, Liu Y, Zhu J, Luo Q, Hui G. Challenges for Obstetricians and the Countermeasures of COVID-19 Epidemic. *Maternal-Fetal Medicine*. 2020. doi: 10.3760/cma.j.issn.2096-6954.2020.0001.
28. Zhou R, Chen Y, Lin C, Li H, Cai X ,Cai Z, Lin G. Asymptomatic COVID-19 in pregnant women with typical chest CT manifestation: a case report. *Chin J Perinat Med*. 2020;**23**(3). Chinese
29. Zhuang S, Guo J, Cao Y, Chen H, Xu D, Li J, Zhang Y. Perinatal novel coronavirus infection: a case report. *Chin J Perinat Med*. 2020;**23**(2): 85-90. Chinese

**Table S3** Quality assessment of case series included in systematic review

| **Study** | **Year** | **Clear criteria for inclusion** | **Condition measured in a standard, reliable way** | **Valid methods used for identification of the condition** | **Consecutive inclusion of participants** | **Complete inclusion of participants** | **Clear reporting of the demographics** | **Clear reporting of clinical information** | **Outcomes of cases clearly reported** | **Clear reporting of the presenting sites/clinics demographic information** | **Statistical analysis appropriate** | **Overall appraisal** |
| --- | --- | --- | --- | --- | --- | --- | --- | --- | --- | --- | --- | --- |
| Breslin (2020)^19^ | 2020 | 1 | 1 | 1 | 1 | 1 | 1 | 1 | 1 | 1 | 1 | Include |
| Hantoushzadeh (2020)^20^ | 2020 | 1 | 1 | 1 | 2 | 1 | 1 | 1 | 1 | 1 | 1 | Included |
| Liu (2020)^21^ | 2020 | 1 | 1 | 1 | 1 | 1 | 1 | 1 | 2 | 1 | 1 | Include |
| Liu (2020)^22^ | 2020 | 1 | 1 | 1 | 1 | 1 | 1 | 1 | 1 | 1 | 1 | Include |
| Liu (2020)^23^ | 2020 | 1 | 1 | 1 | 1 | 1 | 1 | 1 | 1 | 1 | 1 | Include |
| Liu (2020)^24^ | 2020 | 1 | 1 | 1 | 1 | 1 | 1 | 1 | 1 | 1 | 4 | Include |
| Wu (2020)^25^ | 2020 | 1 | 1 | 1 | 1 | 1 | 1 | 1 | 1 | 1 | 1 | Include |
| Yan (2020)^11^ | 2020 | 1 | 1 | 1 | 1 | 1 | 1 | 1 | 1 | 1 | 1 | Include |
| Ferrazzi (2020)^26^ | 2020 | 1 | 1 | 1 | 1 | 1 | 1 | 1 | 1 | 1 | 1 | Include |

**Table S4** Quality assessment of case reports included in systematic review

| **Author** | **Year** | **Demographic characteristic clearly described** | **History clearly described and presented as a timeline** | **Current clinical condition on presentation clearly described** | **Diagnostic tests or assessment methods and the results clearly described** | **Intervention or treatment procedure clearly described** | **Post-intervention clinical condition clearly described** | **Adverse events (harms) or unanticipated events identified and described** | **Provide takeaway lessons** | **Overall appraisal** |
| --- | --- | --- | --- | --- | --- | --- | --- | --- | --- | --- |
| Alonso Diaz (2020)^27^ | 2020 | 1 | 1 | 1 | 1 | 2 | 2 | 2 | 1 | Include |
| Alzamora (2020)^28^ | 2020 | 1 | 1 | 1 | 1 | 1 | 2 | 2 | 1 | Include |
| Buonsenso (2020)^29^* | 2020 | 1 | 2 | 1 | 1 | 1 | 2 | 2 | 1 | Include |
| Gidlof (2020)^30^ | 2020 | 1 | 1 | 1 | 1 | 1 | 1 | 2 | 1 | Include |
| Gonzalez Romero (2020)^31^ | 2020 | 1 | 1 | 1 | 1 | 1 | 3 | 2 | 1 | Include |
| Iqbal (2020)^32^ | 2020 | 1 | 1 | 1 | 1 | 1 | 1 | 4 | 1 | Include |
| Juusela (2020)^33^ | 2020 | 1 | 1 | 1 | 1 | 1 | 1 | 1 | 1 | Include |
| Kalafat (2020)^34^ | 2020 | 1 | 1 | 1 | 1 | 1 | 1 | 1 | 1 | Include |
| Karami (2020)^35^ | 2020 | 1 | 1 | 1 | 1 | 1 | 1 | 2 | 1 | Include |
| Kelly (2020)^36^ | 2020 | 1 | 1 | 2 | 2 | 1 | 1 | 2 | 1 | Include |
| Lee (2020)^37^ | 2020 | 1 | 1 | 1 | 1 | 1 | 1 | 2 | 1 | Include |
| Lowe (2020)^38^ | 2020 | 1 | 1 | 1 | 1 | 1 | 1 | 4 | 1 | Include |
| Schnettler (2020)^39^ | 2020 | 1 | 1 | 1 | 1 | 1 | 1 | 1 | 1 | Include |
| Vlachodimitropoulou Koumoutsea (2020)^40^ | 2020 | 1 | 1 | 1 | 1 | 1 | 1 | 1 | 1 | Include |
| Zamaniyan (2020)^41^ | 2020 | 1 | 1 | 1 | 1 | 1 | 1 | 2 | 1 | Include |
